# Social capital, government guidance and contract choice in agricultural land transfer

**DOI:** 10.1371/journal.pone.0303392

**Published:** 2024-05-09

**Authors:** Linbo He, Jun Huang

**Affiliations:** Hunan Agricultural University, Changsha, HuNan, China; Sichuan Agricultural University, CHINA

## Abstract

This study explores the impact of farm households’ social capital characteristics and local government policies on the selection of farmland transfer contracts in China’s rural industrial revitalization context. Utilizing field research data from 1,979 households in ethnic areas of Hunan Province, this paper constructs an econometric model to assess how farm households’ social capital and local governments’ involvement in rural industrial revitalization influence farmland transfer contract selections. The findings indicate that, lacking government program support, farmers’ social capital significantly affects contract type and duration, but not the rent. Specifically, farmers possessing extensive social capital prefer verbal and short-term contracts (coefficients of 0.525 and 0.643, significant at the 5% level), whereas their influence on rent (coefficient of 2.418, significant at the 5% level) manifests under government program support. These results challenge the conventional theory of farmland transfer contracts and offer substantial empirical support for the development of local government policies in rural industrial revitalization, underlining the critical role of government guidance and social capital in enhancing farmland transfer.

## 1. Introduction

Industrial prosperity is a fundamental requirement of the rural revitalization strategy, with the development of large-scale agriculture or characteristic industries serving as a crucial pathway to achieving rural industrial revitalization. Within the project framework, the development of large-scale agriculture or characteristic industries predominantly depends on agricultural management entities such as "leading enterprises", professional cooperatives, large farmers, or family farms, necessitating a suitable aggregation of agricultural land use [1]. In rural China, the family contract responsibility system constitutes the foundational framework for agricultural land utilization, significantly enhancing the enthusiasm of farmers and promoting agricultural development [2]. With economic and social development, numerous rural young and able-bodied laborers migrate to cities for non-agricultural employment, leading to extensive uncultivated agricultural land and its frequent abandonment. This trend has resulted in fragmented agricultural land use, intensifying the contradiction between the family contract responsibility system and the mode of agricultural production [3]. Consequently, in the rural industrial revitalization process, the transfer of agricultural land, serving as a key land management mechanism, plays a vital role in enhancing the effective utilization of land resources and facilitating the operation of agricultural industrialization [4]. Nevertheless, the effectiveness of agricultural land transfer is significantly influenced by the social capital characteristics of farmers and local government policies. Despite the increasing number of studies, there remains a lack of comprehensive analysis on the impact of these factors on farmland transfer contract choices, particularly in specific socio-cultural contexts. This study seeks to investigate how farmers’ social capital and government guidance influence farmland transfer contract choices in an ethnic region of Hunan Province, aiming to provide empirical support for developing and implementing strategies to promote rural industrial revitalization.

The contract serves as the medium for agricultural land transfer, formalizing and expressing the intent of the transfer, thereby stabilizing expectations, protecting the legal rights of the parties involved, and reducing transaction risks. Contracts can be classified into written and oral, short-term and long-term, as well as low-rent and high-rent agreements. Generally, different contracts exhibit significant variations in transaction costs and benefits. In an ideal market scenario, written, long-term, and high-rent contracts are preferred for stabilizing transaction expectations, minimizing costs, and maximizing market returns [5]. However, in practice, during spontaneous farmland transfers without project support, many transferors opt for verbal, short-term, and rent-free or low-rent contracts, seemingly contravening the principle of rational choice. Conversely, in project-supported farmland transfers, most transferees select written, long-term, and high-rent contracts. The social capital characteristics of farmers in spontaneous farmland transfers and the governmental position and role in project-supported transfers may significantly influence the contractual choices [6]. Within the framework of rural industrial revitalization, farmland transfer is crucial for achieving moderate-scale agricultural development and fostering characteristic industries. While existing research has delved into various farmland transfer contract forms, tenure, and rent options [7–9], there is scant analysis on the motivations behind these choices from the social capital and government guidance perspectives.

This study addresses a crucial gap in the literature by providing a comprehensive analysis of how farmers’ social capital characteristics and local government policies affect contractual choices in farmland transfers, particularly within the context of China’s rural industrial revitalization. Although prior research has examined the impacts of social capital and government roles, it often neglects the interaction between these factors and their collective impact on contract choices in distinct socio-cultural contexts. Utilizing detailed data from 1,979 farming households in a specific ethnic area of Hunan Province, this study employs statistical regression with SUR to comprehensively analyze the impact of social capital and government guidance on contractual choices in agricultural land transfers. Hunan Province, a significant agricultural area in central China, was selected due to its extensive agricultural base, rich cultural resources, particularly in its ethnic regions, which offer a unique social and cultural structure, active agricultural activities, and thus, a rich empirical setting for analyzing the effects of agricultural land transfer policies on the rural economy. Moreover, agricultural land transfer activities in Hunan Province mirror common challenges in Chinese rural areas, including land use efficiency, farm household income growth, and rural social stability. Therefore, by concentrating on Hunan Province, this study seeks to offer insights into the influence of social capital and local government policies on the selection of agricultural land transfer contracts and their role in promoting rural industrial revitalization strategies. Importantly, an in-depth examination of this specific region reveals socio-economic mechanisms that have broad significance for the choice of agricultural land transfer contracts. The detailed localized analysis offers crucial insights into the dynamics of farmland transfers, both in China and in other countries undergoing agricultural transition, particularly in how social capital and government policies collaboratively shape the structure of farmland operations and agricultural development. Consequently, while this study concentrates on an ethnic region in Hunan Province, its findings hold considerable theoretical and practical relevance for comprehending the complexities and multidimensional aspects of contractual choices in agricultural land transfers, aiding in the development of rural industrial revitalization and land management policies both in China and worldwide.

The study is organized as follows: The paper begins with an introduction that outlines the research background and importance of the choice of farmland transfer contracts, emphasizing the role of social capital and government guidance in rural industrial revitalization in China. Following this, the literature review synthesizes previous studies on the impacts of social capital and government guidance on the choice of farmland transfer contracts, providing theoretical support and a foundation for this study. In the section on theoretical basis and hypotheses, specific hypotheses are proposed based on theoretical analysis to explore how social capital and government guidance influence the choice of farmland transfer contracts. The data sources and model section describes the data collection process and the econometric model used. The results and discussion section presents the empirical analysis outcomes and discusses their implications for farmland transfer policies and practices. Finally, the conclusion summarizes the study’s findings and suggests limitations and directions for future research.

## 2. Literature review and commentary

### 2.1 Social capital

Social capital, deriving from the economic concept of "capital," lacks a uniform definition but functions similarly to traditional capital by serving as an input factor in economic production or service activities, enhancing the pace or extent of economic development. Social capital, as an input in economic production or service activities, can be defined from various perspectives: According to some scholars, social capital comprises the structural elements within a social network, such as weak ties, strong ties, or "structural holes." These structural elements, akin to human, financial, and physical capital, enhance the efficiency of economic production or service activities, aiding in the attainment of an individual’s or organization’s goals [10, 11]. The strength of farmers’ linkages often stems from blood relations and geographic proximity. Additionally, it pertains to the frequency of interaction, duration of acquaintance, level of intimacy, and reciprocal exchanges. Some scholars define social capital as the opportunity or ability of individuals or organizations to access resources, such as information, power, or economic assets, based on their position within a social network [12, 13]. In this context, social capital parallels traditional capital but emphasizes the capacity to mobilize resources, such as information, for social action and informed decision-making. For instance, a farmer’s strong connections to leading enterprises, cooperatives, or local governments can facilitate the identification of farmland transfer opportunities. Some scholars view social capital as the trust and norms established through long-term interaction within social networks, which facilitate coordination, reduce transaction costs, and improve the efficiency of economic or service activities [14]. In this perspective, increased trust and reciprocal norms within social networks can lower communication and negotiation costs, reduce fraud, and decrease transaction costs.

In numerous studies, social capital is seen as the resources and support individuals or organizations can mobilize and utilize through their social networks, encompassing information flow, trust building, norm adherence, and social relationship formation [15–18]. Considering the varied interpretations of social capital across different theoretical frameworks, this study adopts a comprehensive definition, viewing social capital as the network of social relationships and resources that farmers can mobilize during farmland transfer. These resources and networks extend beyond direct economic benefits to include trust and norms that facilitate cooperation and transactions. This study posits that social capital plays a pivotal role in the contractual choices for farmland transfer. Specifically, social capital fosters trust between transacting parties, reducing cooperation risks and transaction uncertainties, thereby influencing the form and terms of farmland transfer contracts. Therefore, understanding and assessing social capital’s role in agricultural land transfer behavior is crucial for unveiling the operating mechanisms of the land transfer market.

### 2.2 Types of agricultural land transfer contracts

Agricultural land transfer contracts, as institutional arrangements, encapsulate the transaction willingness of the transferor, implicitly acknowledge the incentives, and aim to provide stable transaction expectations while mitigating transaction risks [19]. These contracts vary primarily in form, duration, and rent, influenced by market conditions and social dynamics.

The form of agricultural land transfer contracts is categorized into written and verbal agreements [20]. In ideal market conditions, written contracts are preferred in real estate transactions. They offer lower costs and risks [21]. Research indicates a preference for verbal agreements in rural China’s farmland transfers. This trend persists even in government-led initiatives for rural industrial revitalization. In such cases, more than half of the transactions are verbal [22, 23]. According to the authors’ field research, in the context of the government’s efforts to promote farmland transfer for rural industrial revitalization, more than 50% of farmland is also in the form of verbal contracts, and although the data are mainly derived from specific ethnic areas in Hunan Province, these findings may be informative for understanding the patterns of farmland transfer in China and even in other regions.

The contract duration affects investment behaviors and land use. Contracts are either short-term or long-term. Long-term agreements foster stable transaction expectations and encourage investment. Short-term contracts often lead to speculative behaviors [24]. Studies show that negotiation difficulty and contract rights stability affect the contract length. Higher negotiation costs usually result in longer-term contracts. Instability in land management rights tends to result in shorter commitments [25, 26]. Despite expectations for more long-term contracts due to idle land from rural-to-urban migration, observations show many short-term contracts in rural revitalization efforts [27]. However, according to the authors’ field research, in rural revitalization practice, there are a large number of agricultural land transfer contracts with irregular, arbitrary or short-term duration. These observations are based on the specifics of the region, but this may reveal more general trends and considerations that apply to the broader context of agricultural management and policymaking.

The rent of agricultural land transfer contracts varies from rent-free or low-rent to high-rent. The rent level mirrors the degree of marketization [28]. Market demand generally dictates high rents. However, transaction costs and property rights stability also influence rent levels. Higher transaction costs and unstable rights lead to higher rents [29]. Social relationships affect the rental terms as well. Transactions among acquaintances or relatives often involve higher rents. In contrast, those between strangers or weakly connected parties tend to have lower rents. Field research in rural revitalization practices reveals an unusual trend. Strong relationships often lead to very low or even zero rent [30]. However, according to the authors’ field research, in rural revitalization practice, there is an inverted phenomenon of farmland transfer rent, fewer normal rent contracts, and very low or even zero rent between strong relationship transferees. Although these findings are based on field research in specific areas, we believe that they can provide insights into contractual choices for farmland transfers in other similar socio-economic environments.

### 2.3 Choice of contract for the transfer of agricultural land

The selection of agricultural land transfer contracts is influenced by a variety of factors like laws, policies, social relations, and the personal characteristics of the transferor. These elements affect the transaction costs of contract governance, shaping the preferred contract type for agricultural land transfers. Contract governance can be categorized into market governance, relationship governance, and hybrid governance, each corresponding to different influencing factors:

Laws or policies significantly impact the choice of agricultural land transfer contracts. The market mechanism’s efficacy depends on property rights protection, freedom of contract, and accountability for faults, along with the stability of industrial, governance, or judicial policies. In a well-functioning market system, the transaction costs for negotiating, fulfilling, and resolving disputes in farmland transfer contracts are lower. Thus, transferors are inclined toward written, long-term, and high-rent contracts for farmland transfers [31].

Social relations play a crucial role in contract selection for agricultural land transfers. Despite the completeness of market mechanisms, contracts remain inherently incomplete, leaving considerable discretion and depending on residual control for issue resolution [32]. This allocation of residual control is partly governed by relational dynamics. In rural China, where market mechanisms are less developed and property rights and contract freedom are limited by non-legal or policy factors, social relations often substitute for market mechanisms. The concept of "differential order pattern" from Fei Xiaotong illustrates that social relations, organized around personal closeness, significantly govern the agricultural land transfer contracts. In such settings, acquaintances may not require written contracts for land transfers, with the duration of tenancy and rent being quite flexible, based on relationship closeness or strength.

Individual characteristics also influence the choice of farmland transfer contracts. Research indicates that the personal traits of farmland transferors, including social demographics like age, gender, education, and occupation, as well as endowment attributes such as information access, problem-solving capabilities, and experience, along with psychological factors like personality, attitudes, values, and emotions, significantly impact their contractual preferences [33–36]. These personal factors shape how transferors approach contract negotiation and agreement, affecting the overall dynamics of farmland transfer contracts.

### 2.4 Literature evaluation

The existing literature and field research indicate a prevalent trend towards verbal, short-term, and low-rent contracts for farmland transfer, contrasting with fewer written, long-term, and high-rent contracts [37, 38]. The explanations provided by institutional economics and the "differential pattern" governance theory for this phenomenon have limitations.

Institutional economics attributes this trend to inadequate protection of property rights, restricted contract freedom, and a low degree of legal enforcement. It suggests that these factors hinder the market mechanism’s role in resource allocation during agricultural land transfers, leading to a predominance of verbal, short-term, and low-rent contracts. However, this explanation seems less convincing as property rights in rural farmland have become relatively clear after years of development. Market transactions are largely free from legal or policy constraints, and disputes can be settled through legal means.

The "differential order pattern" theory, on the other hand, interprets this phenomenon through the lens of social relation strengths. It posits that strong social ties, influenced by obligations or the need to save face, prefer verbal, short-term, and low-rent contracts for land transfers. In contrast, weak social ties, which lack trust, opt for written, long-term, and high-rent contracts [39, 40]. This theory rationalizes the prevalence of verbal and short-term contracts in terms of strong relationships that foster trust and offer internal dispute resolution mechanisms, thus reducing transaction costs. However, this framework falls short in explaining the low-rent aspect, as farmers under normal circumstances wouldn’t accept minimal or no rent purely based on relational ties.

Furthermore, "differential pattern," as a structural functionalism concept, contrasts with the "group pattern" in Western societies. Fei Xiaotong later acknowledged that this perspective focuses more on structure than on the individual, struggling to capture the real emotions, attitudes, or behaviors in decision-making. Ethical considerations play a significant role in shaping decisions.

The authors suggest that while the market mechanism’s completeness and "differential order pattern" governance influence the contractual choices in agricultural land transfer, underlying reasons are more profound. They highlight that the social capital of farmers and government guidance are instrumental in shaping these contractual preferences, suggesting a complex interplay of market dynamics, social structures, and individual attributes in the decision-making process for agricultural land transfers.

## 3. Rationale and assumptions

### 3.1 Social capital and contractual options for agricultural land transfer

Analyzing the agricultural land transfer market as a whole, the supply-demand relationship significantly impacts land rent; rent increases when demand surpasses supply and decreases when supply exceeds demand. From a micro perspective, farmers’ social capital influences agricultural land transfer rent through several pathways: firstly, by aiding in gathering information about land transfers, thus creating more trading opportunities; secondly, by facilitating the identification of better trading partners, leading to more favorable transaction prices; and thirdly, by reducing transaction costs, which enhances trading stability. Despite the influence of farmers’ social capital on land transfer rent, the market’s supply-demand dynamics remain the decisive factor. The prevalence of zero or low rent in land transfer contract choices reflects weak demand for agricultural land, not necessarily strong social connections or a "differential structure" in governance.

Farmers’ social capital, while not a determining factor in land transfer rent, critically influences the contract’s form and duration. In transactions involving farmers, families, and cooperatives, where kinship or community ties exist, social capital is abundant, ensuring rental income and dispute resolution through personal relations or community norms. Often, these parties avoid formal written contracts, preferring verbal agreements based on mutual understanding and trust. However, in contracts between farmers and external entities like leading enterprises, foreign investors, or contractors, where social capital is limited and mutual trust scarce, formal written contracts are the norm. These agreements specify lease periods explicitly to minimize dispute risks and transaction costs related to dispute resolution [41].

Based on these observations, this study posits the following hypothesis:

H1: In the absence of government program guidance, farmers with richer social capital are more likely to engage in verbal and short-duration farmland transfer contracts, which has an insignificant effect on the rent of farmland transfers.

### 3.2 Social capital, government intermediaries and contractual options for agricultural land transfers

In the rural revitalization context, substantial financial transfer payments enter rural areas via industrial projects. Local governments form complex agreements with rural business entities like leading enterprises, cooperatives, or large farming households. These agreements often include financial support for industrial or poverty alleviation projects, with the expectation that these rural business subjects will create interest linkages with impoverished or local farming households. This linkage commonly manifests in land transfers, share dividends, and labor utilization, especially as many able-bodied rural workers migrate for urban employment, leaving agricultural land idle. Consequently, land transfer becomes a preferred method of interest linkage, amplified by government-driven investments in rural revitalization, which in turn expands the demand for and generally increases the rent for transferred farmland.

On the micro-level, however, not all farmland transfers command high rents. The social capital of farm households significantly affects the rental value of their land. Farmers with strong connections to local government staff and rural business entities can access market information more rapidly. Additionally, these entities often prioritize land transfers with local farmers when acquiring industrial projects, suggesting that farmers with more substantial social capital are likely to secure higher rents in these government-facilitated transfers. Since local governments must audit and approve industrial projects, including the farmland transfers involved, the contracts often reflect a combination of written agreements, long-term commitments, and higher rents [42].

Conversely, farmers with limited social capital struggle to access market information and gain support from rural business entities. Their idle land often remains unused or is transferred at minimal or no rent. Based on these observations, the following hypothesis is proposed:

H2: Under government program guidance, farmers with more substantial social capital are more likely to engage in written farmland transfers that include long leases and high rents.

**Fig 1:**
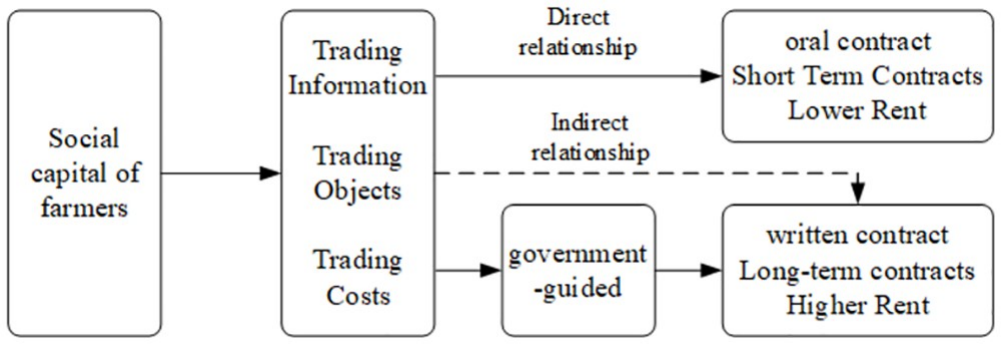
Schematic diagram of the relationship between the role of social capital, government guidance and contractual options for farmland transfer.

In conclusion, the study puts forward two hypotheses, illustrating the interplay between social capital, government intermediary and the choice of farmland transfer contract is shown in the Fig 1.

## 4. Data sources and models

### 4.1 Data sources

The data for this study are derived from a survey conducted on farm households in Hunan Province between 2018 and 2020, focusing on land transfer contracts, project-based systems, and rural industry revitalization. To meet the objectives of rural revitalization, representative counties and cities from East, Central, and West Hunan were chosen, including 33 administrative villages across 10 townships, totaling 1,979 households. This survey spanned various economic levels and cultural backgrounds within Hunan Province, notably in several ethnic areas, thus ensuring the diversity and comprehensiveness of the research data.

Hunan Province was selected for its representative nature and diversity in agricultural production, and its leading role in implementing rural land management policies in China. The detailed examination of 1,979 farming households provided essential data on agricultural land transfer patterns, social capital structure, and the influence of government policies. This study offers insights into the interplay of these factors in the decision-making process for land transfer contracts. Analyzing this field data facilitated a grounded understanding of the land transfer situation and challenges in Hunan Province and more broadly in rural China, laying a robust foundation for developing effective policy recommendations.

Among the surveyed farmers, 1,389 were involved in land transfer activities, with 793 participating in land outflow and 596 in land inflow. The survey aimed to isolate the effects of social capital and government guidance on agricultural land transfer contracts. To achieve this, the questionnaire was structured to first identify participants in land transfer activities. Following affirmative responses, further inquiries determined whether these transfers were facilitated by government programs, allowing for a distinct analysis of transfers with and without government support. The questionnaire also gathered data on individual and environmental characteristics of the farmers. It included specific questions addressing the mode of the farmland transfer contract, the duration of the transfer, and the rental price. This approach was designed to elucidate how social capital and government initiatives influence the various aspects of land transfer agreements.

#### 4.1.1 Social capital and farmland transfer contract selection without government program support

The questionnaire data were meticulously processed and analyzed, leading to the development of a descriptive statistical table focused on social capital and the choices of farmland transfer contracts without the support of government projects. According to Table 1, the farmland transfer agents are categorized into four groups: friends and relatives, farmers within the same village, farmers from other villages, and foreign enterprises. In scenarios lacking government program support, transfers among friends and relatives emerge as the most prevalent, constituting 44.3% of outflows and 51.5% of inflows. This category predominantly opts for verbal contracts, with written contracts comprising only 12.6% and 17.3% of outflows and inflows, respectively. Typically, the lease periods within this group are shorter.

**Table 1:** Social capital and farmland transfer contract selection under the condition of no government program.

Social capital	Percentage of circulation (households) (%)	Written share (households) (%)	Rents ($/mu)	Deadlines (years)	Percentage of circulation (households) (%)	Written share (households) (%)	Rents ($/mu)	Deadlines (years)
disgorge					inflow			
friends and relatives	151/44.3%	10/12.6%	145.3	2.13	143/51.5%	13/15.3%	176.7	2.21
Peasant households in the same village	113/33.1%	13/15.9%	146.1	3.45	61/22.2%	14/16.7%	177.6	3.54
rural outlying villages	43/12.5%	26/32.9%	148.8	4.26	30/10.9%	25/30.4%	180.3	4.57
foreign enterprise	34/9.9%	30/37.8%	150.2	5.69	43/15.5%	31/37.3%	185.6	6.84
(grand) total	341	79			277	83		

Conversely, foreign enterprises, which represent the smallest fraction of transfer agents, tend to favor written contracts and engage in transfers with longer lease periods. Rent levels across the board are generally low, with the lowest rents observed in transfers between relatives—at 145.3 yuan per mu for outflows and 176.7 yuan per mu for inflows. Rents in transfers involving foreign enterprises are slightly higher, at 150.2 yuan per mu for outflows and 185.6 yuan per mu for inflows. These findings preliminarily validate hypothesis 1, indicating that farmers with richer social capital tend to favor verbal contracts and shorter or irregular rental periods for farmland transfers. However, the impact on the rent of farmland is comparatively minimal.

#### 4.1.2 Social capital with government program support, government intermediary and farmland transfer contract selection

Data from questionnaires involving government project support was extracted and categorized to create a descriptive statistical table focusing on social capital under government project support, government guidance, and contractual choices for farmland transfer. In Table 2, friends and relatives are no longer the primary parties in farmland transfers. Instead, farmers within the same village and foreign enterprises emerge as the main parties, accounting for 44.1% and 32.6% of outflows, and 48.2% and 35.7% of inflows, respectively.

**Table 2:** Selection of social capital, government intermediaries and farmland transfer contracts under the condition of having government programs.

Social capital	Percentage of circulation (households) (%)	Written share (households) (%)	Rents ($/mu)	Deadlines (years)	Percentage of circulation (households) (%)	Written share (households) (%)	Rents ($/mu)	Deadlines (years)
disgorge					inflow			
Friends and relatives	32/7.1%	22/68.8%	335.3	3.16	43/13.5%	33/76.7%	341.4	3.19
Peasant households in the same village	153/33.8%	131/85.6%	347.2	3.65	102/32.0%	94/92.2%	357.5	3.78
Rural outlying villages	33/7.3%	32/96.9%	358.1	5.21	31/9.7%	25/80.6%	364.7	5.58
Foreign enterprise	234/51.8%	230/98.3%	360.2	6.71	143/44.8%	132/92.3%	361.5	6.88
(grand) Total	452	416			319	269		

The prevalence of written contracts has significantly increased, with their use in both scenarios exceeding 70%, and among friends and relatives surpassing 50%. Rental periods have generally lengthened, averaging more than three years. Farmland transfer rents have significantly risen, averaging over 350 RMB per mu.

This data initially supports Hypothesis 2: under the project system, government guidance has expanded market demand for farmland transfer, significantly elevating the overall market price for such transfers. Furthermore, it has altered the dynamics of farmers’ social capital, leading to a higher proportion of written contracts and substantially longer rental periods.

### 4.2 Overview of the model

In the same farmland transfer contract selection behavior, the contract mode, contract duration and land rent belong to different aspects of the same choice or decision, which are bound to be correlated and are not suitable for estimation by a system of joint equations, and the systematic estimation of the system of equations can be made by the likelihood of non-correlation method, and the corresponding statistics can be obtained. Based on the research data of 1389 households participating in agricultural land transfer in Hunan Province, this study analyzes the relationship between social capital, government guidance and contractual choices of agricultural land transfer through econometric modeling. Specifically, this paper adopts the Seemingly Unrelated Regression (SUR) model to deal with the potential correlation between the dependent variables, and its standard model is:

$$Y_{j}=X_{j}\beta_{j}+Z_{j}\gamma_{j}+\epsilon_{j},\;j=1,2,\ldots ,M$$ (1)

Where *Y*_*j*_ denotes the dependent variable; *X*_*j*_ denotes the matrix of the main independent variables; *Z*_*j*_ denotes the matrix of the control variables; *β*_*j*_ and *γ*_*j*_ are the parameter vectors; and *ϵ*_*j*_ is the error term.

The seemingly uncorrelated regression model is suitable for situations where the disturbance terms in multiple regression equations may be correlated with each other. In this study, contract mode, contract duration and land rent are used as explanatory or dependent variables, and all three belong to different aspects of the same farmland transfer decision, so their error terms may not be independent. The independent variables are social capital and government guidance, and other related factors are used as control variables to test the hypothesized relationship between social capital, government guidance and contractual choice of agricultural land transfer through statistical regression analysis.

Specifically, the contract mode is divided into verbal contract and written contract, the verbal contract takes the value of 0, and the written contract takes the value of 1; the contract period is filled in freely by the respondents, ranging from 0 to 20, and takes the value of a rational number; the land rent is also filled in freely by the respondents, and takes the value of a rational number. By constructing an econometric regression model and inputting it into Stata software for statistical analysis, in order to obtain more accurate and reliable statistical estimation results, and then to verify the hypothesized relationship between social capital, government guidance and the choice of contract for agricultural land transfer.

### 4.3 Description of variables

The types and definitions of the dependent, independent and control variables are detailed in Table 3 and explained in detail below:

**Table 3:** Variable types and definitions.

Variable type		Variable Definition	Average value	(statistics) Standard deviation	Average value	(statistics) Standard deviation
			transfer out		shift to	
Implicit variable	contract terms	Oral contract = 0, written contract = 1	0.310	0.438	0.181	0.394
	contract duration	Unit: Year	2.140	0.892	1.85	0.843
	Agricultural land rental	Unit: yuan/acre	245.30	385.14	325.21	331.54
Independent variable	social capital	Family and friends = 1, residents of the same village = 2, residents of other villages = 3, foreign enterprises = 4	2.140	1.204	2.240	0.850
	government intermediary	No project support = 0, with project support = 1	0.381	0.421	0.514	0.214
Control variable	(a person’s) age	Unit: years	54.214	12.653	52.142	9.325
	educational attainment	Illiterate = 1, Elementary school = 2, Middle school = 3, High school = 4, University and above = 5	2.623	1.254	2.364	1.543
	Sources of household income	Farming-based = 1, working outside the home = 2	1.524	1.024	1.621	1.251
	Number of insured persons in the family	Unit: persons	2.054	1.201	2.635	1.214
	Flow area	Unit: acres	5.214	2.254	6.524	2.341
	water conservancy conditions	Bad = 1, Fair = 2, Good = 3	1.952	1.024	2.541	1.201
	transport condition	Bad = 1, Fair = 2, Good = 3	2.511	1.254	2.547	1.263

#### 4.3.1 Social capital

In Chinese rural society, social relations are rooted in connections such as blood, geographical proximity, or academic affiliations. The social capital of farmers is often navigated through administrative ethics, where the abundance of social capital can lead to behavioral constraints influenced by concerns over reputation or the need to reciprocate favors. The method for measuring social capital is well-documented, with different metrics applicable based on the specific type of social capital being assessed [43].

In this study, the intensity of the relationship is utilized as a direct indicator of a farmer’s social capital. The measurement scale categorizes connections with family and friends as 1, indicating the strongest social bonds and hence the highest social capital. Residents from the same village are rated as 2, reflecting a lesser degree of social capital compared to immediate family and friends. Relationships with individuals from different villages are assigned a value of 3, indicating weaker social ties. Finally, interactions with foreign enterprises are marked as 4, representing the least strong relationship and thus the lowest level of social capital. Essentially, the higher the numerical value, the weaker the relationship and, consequently, the lesser the social capital.

#### 4.3.2 Government guidance

During the rural revitalization initiative, the government’s role includes overseeing the progress and results of industrial projects managed by rural business entities. For process supervision, authorities establish agricultural land transfer centers or platforms, requiring that land transfers occur through these channels. This approach aids in monitoring the activities related to land transfers. Outcome supervision, on the other hand, focuses on evaluating the completion and success of projects. It mandates that rural business entities provide written reports or project outcomes for governmental evaluation. In this context, the farmland transfer contract emerges as an essential document for review, underscoring its significance in the official oversight process.

In cases of agricultural land transfers that proceed without government project support, the government’s supervisory role is less prescriptive. Transferors are not obliged to adhere to strict supervision but are encouraged to voluntarily register their transfer agreements with the designated platform or center, ensuring a record of the transaction. To quantify the influence of government program support on agricultural land transfers, a binary value system is employed: transfers under government support are marked with a value of 1, signifying their compliance and formal oversight, whereas those occurring independently of government programs are given a value of 0, indicating the absence of direct governmental involvement in the supervisory process.

#### 4.3.3 Individual characteristics

A substantial body of literature demonstrates that the individual social characteristics of farm households significantly impact the contractual choices for farmland transfer. Research indicates that older farm households and those with lower labor capacity tend to transfer farmland out rather than in, though its impact on contractual choices remains uncertain [44]. Higher education levels in farm households, which correlate with better knowledge, ability, or experience, are likely to lead to a departure from traditional ethical concepts like favors or face-saving, and increase the preference for written, long-term, and high-rent contracts.

If a farm household’s primary income is derived from agriculture, this signifies the importance of stable agricultural income, making them more inclined to choose written, long-term, and high-rent contracts. Furthermore, with the enhancement of the rural social security system, the role of land as social security diminishes, and farmers’ participation in insurance schemes significantly influences their choices of farmland contracts.

To summarize, this study selects age, education level, primary source of family income, and the number of insured individuals as control variables influencing the contractual choices of farmland transfer.

#### 4.3.4 Environmental characteristics

Research indicates that environmental factors significantly influence contract choices in agricultural land transfers. In mountainous regions, characterized by small and scattered farmland plots, the land is often unsuitable for large-scale operations by rural business entities, and market demand is typically low. In such areas, farmers may place less emphasis on the specifics of contract forms or lease durations and may have lower rent expectations. Conversely, in areas where farmland is expansive and conducive to large-scale operations, market demand is higher. Here, farmers prioritize income security, showing a marked preference for specific contract types and lease periods, with correspondingly higher rent expectations [45].

Further studies suggest that infrastructure aspects like transportation and water conservancy facilities also play a crucial role. Improved transportation and irrigation conditions enhance the attractiveness of land for transfer, influencing farmers’ decisions regarding contract form, lease duration, and rent levels [46]. These environmental and infrastructural factors thus critically shape the contractual dynamics in the agricultural land transfer market.

## 5. Results and discussion

### 5.1 Results and analysis

To validate Hypothesis 1, data on the outflow and inflow of agricultural land transfer contracts, conducted without government program support, were merged for statistical analysis. The variable representing government intermediation was set to 0. Using the seemingly uncorrelated regression model with contract mode, contract duration, and land rent as the dependent variables, and social capital as the independent variable (along with other control variables), the analysis was conducted. The results are displayed in Table 4, showing social capital as a significant influencer in the choice of agricultural land transfer contracts when government intermediation is absent.

**Table 4:** Social capital and farmland transfer contract choice.

Variant	Contract terms		Contract duration		Land rent	
	ratio	Z-value	ratio	Z-value	ratio	Z-value
Social capital	-0.314**	2.532	-0.432**	1.493	1.329	2.324
(a person’s) Age	-0.295**	-2.329	0.692	1.372	0.054	0.862
Educational attainment	0.013*	1.329	0.539**	2.218	0.218**	2.315
Sources of household income	-0.403**	-1.149	-0.531	-0.835	-0.478	-1.129
Number of insured Persons in the family	-0.854	-1.478	0.047	1.365	-0.376	-1.964
Flow area	0.014**	2.842	0.001*	1.458	0.010*	1.384
Water conservancy conditions	0.583**	2.475	0.756	0.578	0.148	1.647
Transport condition	0.015**	3.340	0.387	1.129	0.022	0.231
Parameter term	6.243***	1.479	2.592	2.341	-0.025*	-1.834
Goodness of fit (R^2^)	0.311		0.323		0.301	

Note:

*, ** and *** indicate significance at the 10%, 5% and 1% levels, respectively.

The findings from Table 4 reveal that an increase in social capital correlates with a preference for verbal and short-term contracts, as indicated by the negative coefficients of -0.314 and -0.432, respectively, both significant at the 5% level. This trend suggests that, devoid of government influence, farmers rely more on their social networks, leveraging these relationships to decrease transaction costs and risks. This reliance likely stems from the ability of social networks to alleviate information asymmetry, foster trust, and reduce monitoring expenses.

Interestingly, while social capital markedly affects the form and duration of contracts, its impact on land rent is not significant (with a coefficient of 1.329, not significant at the 10% level). This indicates that in environments with strong social ties, farmers prioritize flexibility and relational ease over financial gains from rent. It underscores the role of social relationships in the economic decisions of rural communities, where non-economic factors can outweigh monetary considerations.

The statistical model’s goodness of fit for contract form, contract duration, and land rent all exceed 0.3, highlighting the substantial influence of social capital on contractual choices in agricultural land transfers. Therefore, Hypothesis 1 is substantiated: in the absence of government program guidance, farmers with more substantial social capital are inclined towards verbal and short-term land transfer contracts, with negligible impact on the rent derived from these transfers.

To test Hypothesis 2, data on both inflow and outflow of agricultural land transfer contracts with government program support are amalgamated and analyzed, with the government intermediary assigned a value of 1. Utilizing the seemingly unrelated regression model, with contract mode, contract duration, and land rent as dependent variables, and social capital, government intermediary, and their interaction as independent variables, along with control variables, the results obtained are presented in Table 5.

**Table 5:** Social capital, government intermediaries and contractual options for farmland transfer.

Variant	Contract terms		Contract duration		Land rent	
	ratio	Z-value	ratio	Z-value	ratio	Z-value
Social capital	0.525**	1.721	0.643**	2.384	2.418**	1.434
Government intermediary	0.871***	2.134	1.542**	1.782	0.862**	2.110
Social capital* Government intermediary	0.512*	1.463	0.231*	1.475	0.039*	1.493
(a person’s) age	-0.483**	-2.284	0.629	1.385	0.052	0.234
Educational attainment	0.034*	1.683	0.394**	2.395	0.473**	2.575
Sources of household income	-0.398**	-1.189	-0.549	-0.498	-0.341	-1.483
Number of insured persons in the family	-0.573	-1.043	0.032	1.846	-0.954	-1.236
Flow area	0.045**	2.375	0.001*	1.123	0.010*	1.211
Water conservancy conditions	0.859**	2.232	0.494	0.245	0.439	1.245
Transport condition	0.036**	3.492	0.493	1.583	0.032	0.532
Parameter term	6.398***	1.209	2.493	2.127	-0.042*	-1.743
Goodness of fit (R^2^)	0.337		0.304		0.327	

Note:

*, ** and *** indicate significance at the 10%, 5% and 1% levels, respectively.

With government program support, this study sheds light on how social capital and government intermediaries influence contractual choices for farmland transfer. Notably, farmers with substantial social capital are more inclined to opt for written, long-term, and high-rent contracts under government program support (coefficients of 0.525, 0.643, and 2.418, respectively, all significant at the 5% level). This phenomenon may reflect the enhanced sense of security and expected stability provided by government guidance, encouraging farmers to adopt more formal and enduring contract forms. The significant role of the government intermediary in influencing contract modality, duration, and rent is underscored by coefficients highly significant at the 1% level, suggesting that government interventions have redefined and potentially strengthened the role of social capital in a supportive context. The interaction between social capital and government intermediation is also noteworthy, with significant effects on contractual modality, tenure, and land rent at the 10% level. This interaction indicates that under government guidance, social capital not only alters the direction of its influence on contractual choices but also amplifies its impact. These findings emphasize the role of government guidance in the agricultural land transfer market, particularly in promoting more formal and long-term contract choices. They also reveal the complexity of how government policies interact with social capital in rural industrial revitalization efforts.

In conclusion, Hypothesis 2 is confirmed: under government program guidance, farmers with richer social capital tend to prefer written contracts for farmland transfer with longer lease terms and higher rents.

To assess the robustness of the study’s findings, group regression was employed to further examine the consistency of social capital and government guidance impacts on farmland transfer contract choices. Given Hunan Province’s geographic and economic heterogeneity, the research classified samples by regions (Xiangdong, Xiangzhong, and Xiangxi) to investigate the potential influence of geographic areas on contract decisions. Farm households in these regions encounter varied socioeconomic environments, potentially affecting their social capital development and responsiveness to governmental policies.

The group regression outcomes, as displayed in Table 6, demonstrate model goodness-of-fit (R2) values of 0.446, 0.421, and 0.419 respectively. These figures suggest the model’s competent explanation of the data across different regions, signifying an effective portrayal of the variables’ interrelations. Table 7 shows a slight decrease in model explanatory power with R2 values of 0.407, 0.394, and 0.3617, particularly noting a reduced explanatory capacity in the central Xiangzhong region compared to the eastern part.

**Table 6:** Regression results of Xiangdong region.

Variant	contract terms		contract duration		land rent	
	ratio	Z-value	ratio	Z-value	ratio	Z-value
social capital	0.416**	1.632	0.576**	2.186	1.762**	1.013
government intermediary	0.762***	1.986	1.371*	1.662	0.784**	1.986
(a person’s) age	-0.355**	-2.163	0.597*	1.110	0.046	0.172
educational attainment	0.022**	1.137	0.382**	2.105	0.263**	2.188
Sources of household income	-0.376*	-1.204	-0.552	-0.423	-0.379	-1.406
Number of insured persons in the family	-0.562	-1.001	0.024	1.808	-0.887	-1.003
Flow area	0.022*	2.162	0.002*	1.109	0.006*	1.204
water conservancy conditions	0.765**	2.119	0.424	0.206	0.237	1.086
transport condition	0.024*	3.241	0.386	1.222	0.168	0.504
parameter term	5.784***	1.082	2.162	2.008	-0.028*	-1.386
Goodness of fit (R^2^)	0.446		0.421		0.419	

**Table 7:** Regression results of Xiangzhong region.

Variant	contract terms		contract duration		land rent	
	ratio	Z-value	ratio	Z-value	ratio	Z-value
social capital	0.567**	1.867	0.683**	1.983	2.519**	1.861
government intermediary	0.883***	2.424	1.786**	1.920	0.913**	2.249
(a person’s) age	-0.301**	-1.982	0.616*	1.306	0.059	0.186
educational attainment	0.017**	1.108	0.416**	2.213	0.277**	2.204
Sources of household income	-0.427*	-1.101	-0.513	-0.427	-0.295	-1.407
Number of insured persons in the family	-0.426	-0.986	0.127	1.657	-0.841	-1.333
Flow area	0.106**	1.684	0.003*	1.276	0.006*	1.093
water conservancy conditions	1.026**	2.149	0.586	0.251	0.467	1.261
transport condition	0.041**	3.501	0.519	1.607	0.044	0.537
parameter term	6.414***	1.217	2.509	2.116	-0.037*	-1.528
Goodness of fit (R2)	0.407		0.394		0.3617	

In Table 8, the R2 values are 0.406, 0.391, and 0.437, indicating that in western Hunan, the model’s data explanation capability is on par with eastern and central regions, with a marginally better explanation of land rent. Although there is a regional variance in the specific impact strength of social capital and government guidance, the overarching trend aligns with the full-sample estimation outcomes. Social capital and government guidance consistently facilitate the contractual decision-making in agricultural land transfers across regions.

**Table 8:** Regression results of Xiangxi region.

variant	contract terms		contract duration		land rent	
	ratio	Z-value	ratio	Z-value	ratio	Z-value
social capital	0.420**	1.651	0.582**	2.210	1.781**	1.024
government intermediary	0.771***	2.008	1.386*	1.680	0.784**	1.986
(a person’s) age	-0.359**	-2.189	0.604*	1.127	0.051	0.164
educational attainment	0.036**	1.148	0.394**	2.173	0.281**	2.196
Sources of household income	-0.382*	-1.267	-0.571	-0.439	-0.381	-1.512
Number of insured persons in the family	-0.444	-1.197	0.036	1.473	-0.901	-1.067
Flow area	0.027**	2.158	0.004*	1.123	0.003*	1.168
water conservancy conditions	0.682**	2.109	0.406	0.217	0.207	1.094
transport condition	0.018*	3.162	0.357	1.203	0.177	0.516
parameter term	5.627***	1.064	2.118	2.012	-0.034*	-1.349
Goodness of fit (R^2^)	0.406		0.391		0.437	

This regional analysis confirms the findings’ cross-regional applicability, thereby reinforcing the study’s credibility and the robustness of its results. The persistence of these trends across different geographic areas attests to the positive influence of social capital and government guidance on the contract selection process in agricultural land transfers.

To address potential endogeneity bias stemming from reverse causality and unobservable variables, an endogeneity test is essential in this research. For this purpose, government project support is employed as an instrumental variable (IV) for social capital. Government support can be distinctly identified as a binary variable, indicating whether a farmer has received support from government programs. This determination relies on existing administrative records and can be quantitatively assessed based on the extent or magnitude of government program support, reflected in metrics like the volume of government funding or the scope and quality of services provided.

The method of IV-2SLS (Two-Stage Least Squares) regression is utilized to address these concerns. In the first stage, government program support is used to predict the levels of social capital, hypothesizing that government intervention indirectly influences contract choices through its impact on social capital formation. In the second stage, the predicted social capital from the first stage is used to analyze its effect on the contractual choices in farmland transfers. This two-step approach helps to isolate the exogenous variation in social capital that is attributable to government support, thereby reducing the bias in estimating the impact of social capital on contract selection.

The empirical results, presented in Table 9, provide a refined assessment of how social capital, influenced by government program support, affects the choices of farmland transfer contracts. This analysis not only enhances the reliability of the findings but also helps in understanding the complex interplay between government policies, social capital, and contractual behaviors in the agricultural land transfer market.

**Table 9:** Instrumental variable approach: Results of regression solution for government program support instead of government guidance.

	Phase I	Phase II		
	government-led	contract terms	contract duration	Agricultural land rental
Government program support	0.005***	2.936***	19.632***	31.201***
Controls	YES	YES	YES	YES
(a person’s) age	YES	YES	YES	YES
educational attainment	YES	YES	YES	YES
Sources of household income	YES	YES	YES	YES
Number of insured persons in the family	YES	YES	YES	YES
Flow area	YES	YES	YES	YES
water conservancy conditions	YES	YES	YES	YES
transport condition	YES	YES	YES	YES
N	1389	1389	1389	1389
K-Prk LM		51.632***	51.632***	51.632***
K-Prk Wald F		52.114*** [15.26]	52.114*** [15.26]	52.114*** [15.26]

Note: Critical values for the 10% significance level of the F-test for identification of weak instrumental variables are in square brackets.

The results presented in Table 9 provide robust statistical support for the methodology used in the study. The K-PrkLM statistic of 51.632 strongly rejects the null hypothesis of instrumental variable under-identification at the 1% significance level, indicating that the chosen instrumental variable, government project support, is valid and properly identified in the model. This is further corroborated by the K-Prk Wald F statistic of 52.114, which surpasses the threshold of 15.26 at the 10% significance level for the weak identification test, ruling out the concern of weak identification for the instrumental variable. In the first stage of the analysis, a significant positive relationship between government guidance and agricultural land transfer was established, thus verifying the correlation of the instrumental variable with the explanatory variable. This relationship confirms that government guidance is an appropriate instrument for social capital in the context of agricultural land transfer. Moving to the second stage of the analysis, after accounting for endogeneity between government guidance and agricultural land transfer, the coefficients remain positive and are statistically significant at the 1% level. This finding solidifies the assertion that government guidance plays a significant role in facilitating agricultural land transfers. The positive and significant coefficients indicate that, beyond just being correlated, government guidance actively promotes the execution of agricultural land transfer contracts, confirming the instrumental variable’s efficacy and the model’s overall reliability in capturing the nuanced dynamics of the agricultural land transfer market.

In summary, this study elucidates the effects of social capital and government guidance on the choice of farmland transfer contracts and delineates their interaction mechanisms across various contexts. In the absence of government program support, it was found that social capital significantly heightened farmers’ propensity to opt for informal (verbal) and flexible (short-term) contracts. This reflects the fact that, in the absence of government guidance, farmers are more reliant on social networks to mitigate transaction costs and risks. However, in the context of government program support, the impact of social capital undergoes a significant change. Within this framework, an increase in social capital led to a heightened preference for formal (written), long-term, and high-rent contracts. This suggests that government support engendered more security and anticipated stability among farmers, prompting a shift towards more formal and long-term contractual forms. Moreover, the pivotal role of the government intermediary and its interaction with social capital further amplify this effect, unveiling the decision-making process in the choice of farmland transfer contracts under the synergistic effect of government guidance and social capital.

## 6. Discussion

Prior studies have examined the characteristics of "farmers" and "traditional agriculture" within China’s market structure, noting changes in the Chinese social system [47]. In the context of the imbalance between supply and demand in the agricultural land transfer market, the abundance of social capital leads to a greater tendency to form verbal agreements and short-term contracts between the two parties. However, an abundance of social capital does not augment the rental level of agricultural land transfers, which remain generally constrained by market demand, while significantly influencing the outflow and inflow of rural land transfers [48–50]. This paper investigates the relationship between social capital, government guidance, and farmland transfer contract choice. It was found that social capital significantly affects the choice of farmland transfer contracts, with greater social capital correlating with a preference for verbal and short-term contracts. In the context of government program support, the influence of social capital and government guidance on farmland transfer contract choice becomes more pronounced. Additionally, it was observed that the impact of social capital on farmland transfer rent is not significant, whereas government guidance significantly affects farmland transfer rent.

Our study systematically explores the impact of research findings on the complexity and dynamics of agricultural land transfer markets. We provide new perspectives on how social capital and government guidance significantly affect the choice of farmland transfer contracts, highlighting the need for nuanced policy approaches in this area. The findings suggest that policy formulation should consider the differential impacts of social capital and government intervention on land transfer decisions. Specifically, policies should promote the effective use of social capital in rural communities and ensure that government programs support sustainable and equitable development in land transfer markets. The phenomena of verbal agreements, short-term commitments, and low rents in the contractual choices for agricultural land transfers do not constitute irrational behavior by farmers. Given that market demand for agricultural land is weak, farmers’ preference for verbal agreements, short-term, and low-rent transfers is the optimal choice, leveraging their social capital advantages. To promote written, long-term, and high-rent contracts for farmland transfers, policymakers should consider the positive impact of social capital and work to build strong community networks and trust mechanisms to facilitate the efficient use and transfer of farmland resources. The Government should support the development of farmers’ mutual aid organizations and cooperatives, and promote information exchange and resource sharing among farmers through training and construction activities. These activities could include agricultural technology training, market information-sharing meetings, and seminars on cooperative business management. Meanwhile, the government needs to enhance farmers’ trust in the farmland transfer process by ensuring transaction transparency and fairness, and, if necessary, further strengthen the legal framework to protect farmers’ rights and interests.

Nevertheless, this study has limitations. Primarily, its research sample is confined to the ethnic areas of Hunan Province, potentially restricting its scope. Second, the findings may be influenced by other factors, such as market supply and demand, and farmers’ personal characteristics. Therefore, future research should broaden the sample scope and consider more influencing factors to more comprehensively reveal the relationship between social capital, government guidance, and the contractual choice of farmland transfer.

## 7. Conclusion

This study elucidates the pivotal roles of social capital and government guidance in the choice of farmland transfer contracts through a comprehensive analysis of field research data from 1,979 farm households in the ethnic areas of Hunan Province. The empirical findings indicate that in the absence of government program guidance, farmers’ social capital characteristics significantly affect their propensity to opt for verbal and short-term contracts. This phenomenon underscores the significance of social networks in rural communities and their impact on economic decisions. Conversely, under government program guidance, farmers with substantial social capital tend to favor written, long-term, and high-rent contracts, highlighting the crucial role of government guidance in altering the behavioral patterns of agricultural land transfer markets. The findings of this study provide new perspectives for understanding the complexity and dynamics of agricultural land transfer markets, revealing the dual roles of social capital and government behavior in the contractual choices of farmland transfer and showing how social and political factors jointly shape economic behavior in the strategy of rural industrial revitalization. These findings not only enrich the theoretical framework on farmland transfer and social capital but also point the way for future research, particularly in exploring the impact of the interaction between social capital and government policies on agricultural and rural development.

In summary, this research provides novel insights into the contractual choices for agricultural land transfers, offering substantial theoretical and empirical support for comprehending social and political factors in rural economic development.

## Supporting information

S1 Data (DOCX)
