# Supplementary material for: Social capital, government guidance and contract choice in agricultural land transfer
Source: PLoS One. 2024 May 9;19(5):e0303392. doi: 10.1371/journal.pone.0303392 (PMC11081326; doi:10.1371/journal.pone.0303392)
Supplement: S1 Data — (DOCX) [file pone.0303392.s001.docx]

| Variable type | | variable assignment | average value | (statistics) standard deviation | minimum value | maximum values | average value | (statistics) standard deviation | minimum value | maximum values |
| --- | --- | --- | --- | --- | --- | --- | --- | --- | --- | --- |
|  |  |  | transfer out | |  |  | shift to | |  |  |
| explanatory variable | contract terms | Oral contract = 0, written contract = 1 | 0.310 | 0.438 | 0 | 1 | 0.181 | 0.394 | 0 | 1 |
|  | contract duration | Unit: Year | 2.140 | 0.892 | 0.1 | 4.5 | 1.85. | 0.843 | 0.4 | 5 |
|  | Agricultural land rental | Unit: yuan/acre | 245.30 | 385.14 | 100 | 401 | 325.21 | 331.54 | 120 | 582 |
| explanatory variable | social capital | Family and friends = 1, residents of the same village = 2, residents of other villages = 3, foreign enterprises = 4 | 2.140 | 1.204 | 1 | 4 | 2.240 | 0.850 | 1 | 4 |
|  | government intermediary | No project support = 0, with project support = 1 | 0.381 | 0.421 | 0 | 1 | 0.514 | 0.214 | 0 | 1 |
| control variable | (a person's) age | Unit: years | 54.214 | 12.653 | 19 | 74 | 52.142 | 9.325 | 24 | 81 |
|  | educational attainment | Illiterate = 1, Elementary school = 2, Middle school = 3, High school = 4, University and above = 5 | 2.623 | 1.254 | 1 | 5 | 2.364 | 1.543 | 1 | 5 |
|  | Sources of household income | Farming-based = 1, working outside the home = 2 | 1.524 | 1.024 | 1 | 2 | 1.621 | 1.251 | 1 | 2 |
|  | Number of insured persons in the family | Unit: persons | 2.054 | 1.201 | 0 | 7 | 2.635 | 1.214 | 0 | 7 |
|  | Flow area | Unit: acres | 5.214 | 2.254 | 0.5 | 9.3 | 6.524 | 2.341 | 0.7 | 11.4 |
|  | water conservancy conditions | Bad = 1, Fair = 2, Good = 3 | 1.952 | 1.024 | 1 | 3 | 2.541 | 1.201 | 1 | 3 |
|  | transport condition | Bad = 1, Fair = 2, Good = 3 | 2.511 | 1.254 | 1 | 3 | 2.547 | 1.263 | 1 | 3 |
